# DSPP-MMP20 gene silencing downregulates cancer stem cell markers in human oral cancer cells

**DOI:** 10.1186/s11658-018-0096-y

**Published:** 2018-07-11

**Authors:** Nikolaos G. Nikitakis, Ioannis Gkouveris, Jaya Aseervatham, Kelvin Barahona, Kalu U. E. Ogbureke

**Affiliations:** 1 Department of Diagnostic and Biomedical Sciences, University of Texas Health Sciences Center at Houston School of Dentistry, 7500 Cambridge Street, Houston, TX 77054 USA; 2 Department of Oral Pathology and Medicine, School of Dentistry, University of Athens, Athens, Greece

**Keywords:** Cancer stem cells, Cancer stem cell markers, Oral Cancer, OSCC, MMP20, DSPP, Cisplatin

## Abstract

**Background:**

Recent findings indicate that dentin sialophosphoprotein (DSPP) and matrix metalloproteinase (MMP) 20 interact in oral squamous cell carcinoma (OSCC). The objective of this study was to determine the effects of DSPP/MMP20 gene silencing on oral cancer stem cell (OCSC) markers.

**Methods:**

The expression of well-established OCSC markers: ABCG2; ALDH1; CD133; CD44; BMI1; LGR4, and Podoplanin in DSPP/MMP20-silenced OSCC cell line, OSC2, and controls were assayed by western blot (WB), and flow cytometry techniques. The sensitivity of OSC2 cells to cisplatin following DSPP/MMP20 silencing was also determined.

**Results:**

DSPP/MMP20 silencing resulted in downregulation of OCSC markers, more profoundly ABCG2 (84%) and CD44 (81%), following double silencing. Furthermore, while treatment of parent (pre-silenced) OSC2 cells with cisplatin resulted in upregulation of OCSC markers, DSPP/MMP20-silenced OSC2 cells similarly treated resulted in profound downregulation of OCSC markers (72 to 94% at 50 μM of cisplatin), and a marked reduction in the proportion of ABCG2 and ALDH1 positive cells (~ 1%).

**Conclusions:**

We conclude that the downregulation of OCSC markers may signal a reduction in OCSC population following MMP20/DSPP silencing in OSCC cells, while also increasing their sensitivity to cisplatin. Thus, our findings suggest a potential role for DSPP and MMP20 in sustaining OCSC population in OSCCs, possibly, through mechanism(s) that alter OCSC sensitivity to treatment with chemotherapeutic agents such as cisplatin.

**Electronic supplementary material:**

The online version of this article (10.1186/s11658-018-0096-y) contains supplementary material, which is available to authorized users.

## Introduction

Oral squamous cell carcinoma (OSCC) is the sixth most common malignancy worldwide with more than 500,000 new cases reported and 300,000 resultant deaths annually [1]. OSCCs also account for over 90% of head and neck squamous cell carcinoma (HNSCC) [1]. Surgery with or without adjuvant radiotherapy remains the mainstay of treatment of OSCCs, while currently available chemotherapeutic agents have been of limited success. As a result, the 5-year survival rate for OSCCs patients remained unchanged at 50–55% from nearly five decades ago. This is in spite of advances in new treatment modalities [2–4]. This bleak outlook underscores the urgency for increasing our understanding of the mechanisms of tumor development, progression, and resistance to treatment. It also raises the urgency to identify effective therapeutic strategies and targets to improve survival in OSCC patients.

In recent years, several studies have reported the presence of a small population of cancer stem cells (CSCs) in solid tumors, including OSCCs [5]. This peculiar subpopulation exhibits high tumorigenic potential and is considered to contribute to, if not entirely responsible for, antineoplastic treatment failures [6]. Specifically, CSCs possess several features associated with an aggressive malignant behavior, including high self-renewal capacity and differentiation potential, elevated tumorigenicity, and increased resistance to therapy [5]. The resistance of CSCs to chemotherapy has been suggested to be due to their ability for intake and excretion of various drug substrates related to cellular metabolism, thereby contributing to their survival and chemoresistance [6].

Various proteins have emerged as useful biomarkers of CSCs. Notable among these are CD34, CD133, CD24, CD44, CD29, CD31, aldehyde dehydrogenase (ALDH), B cell-specific Moloney murine leukemia virus integration site 1 (BMI1), podoplanin (PDPN), leucine-rich repeat-containing G-protein-coupled receptors LGR5 and LGR4, and ATP binding cassette transporter 2 (ABCG2) [7–12]. In consequence, these markers remain targets for novel therapeutic strategies aimed at effectively diminishing CSC subpopulation in various cancers [5, 9]. CD44, CD133, BMI1 and ALDH are considered frontline markers for detection of CSCs in OSCC, and other head and neck squamous cell carcinomas (HNSCCs) [5, 13–15]. This knowledge is engendering efforts to decipher the significance of these designated CSC markers in the biology of OSCCs.

Over the past decade, we and others have reported the upregulation of dentin sialophosphoprotein (DSPP), a member of the small integrin binding ligand n-linked glycoproteins (SIBLING) family of extracellular matrix proteins in several human cancers, including OSCCs [16–19]. With respect to OSCC, DSPP expression correlated with tumor aggressiveness and prognosis, including tumor recurrence at primary sites [17, 18]. Furthermore, DSPP silencing in OSCC cell line decreased notable hallmarks of oral tumorigenesis, such as cell viability, invasion and migration, colony-formation, G0/G1 cell cycle arrest, while increasing tumor cell sensitivity to cisplatin-induced apoptosis [20].

Our previous reports also have established that a number of matrix metalloproteinases (MMPs), known to be upregulated in cancers, including OSCC, bind and interact with specific MMPs in biochemical and biologic systems: MMP2 with BSP; MMP3 with OPN; MMP9 with DMP1; and MMP20 with DSPP [17, 18, 21–23]. Notably, in OSCC cells, there is a 9-fold enrichment of DSPP at the MMP20 promoter proximal elements suggesting a regulatory role in MMP20 transcription [23]. Although significant insights on the role of MMP2, MMP3, and MMP9 in OSCCs and other cancers have been gained, the full functional/mechanistic role of DSPP-MMP20 partnering and interaction in the biology of OSCC and other cancers is just beginning to be explored.

The aim of this study was to investigate the effects of DSPP/MMP20 silencing on OCSCs subpopulations in OSCC by evaluating the relative expression of known CSC markers (CD44, CD133, ABCG2, ALDH1, PDPN, BMI1 and LGR4) in parent and DSPP/MMP20 silenced OSCC cell line, OSC2. Furthermore, the dose-dependent effects of cisplatin on OCSC cells in parent and DSPP/MMP20 silenced OSC2 cells were analyzed.

## Methods

### Human cell lines and culture conditions

The *study was approved* by the University of Texas Health Science Center-Houston’s Institutional Review Board for all experimental procedures including human tissue samples and cell lines.

Through our previous studies using various OSCC cell lines, we have validated the OSCC cell line, OSC2, as a model cell line for investigating SIBLING/MMP interaction [23]. For the present study therefore, experiments were carried out on the human OSCC cell line, OSC2, obtained from American Type Culture Collection (ATCC; Manassas, VA, USA). We have recently validated this and other cell lines in our laboratory. As is routine, cells were cultured as monolayer in DMEM/F12 medium containing 10% FBS (Invitrogen, Carlsbad, CA) supplemented with 1% Penicillin/Streptomycin and 500 ng/ml Hydrocortisone (Sigma Aldrich, St. Louis, MO). Cell culture was maintained in the presence of 5% CO_2_ humidified air at 37 °C. For shRNA stable clones (gene-silenced cells), medium containing 4 mg/ml of puromycin (cat # sc-108,071; Santa Cruz Biotech) was used in place of normal medium. Culture medium with puromycin was replaced every 2–3 days.

### DSPP and MMP20 silencing

*MMP20 shRNA (h)* lentiviral particle (cat #sc-*41,561-*V) and *DSPP shRNA(h)* lentiviral particle (cat #sc-40,500-V) were purchased as transduction-ready pools of 3 target-specific constructs encoding 19–25 nt (plus hairpin) shRNAs designed to silence MMP20 and DSPP genes, respectively. A transfection-ready copGFP control Plasmid (cat # sc-108,083) is a lentiviral vector encoding copGFP fluorescent protein in mammalian cells. This was used to assess the delivery and transfection efficiency of the shRNA lentiviral construct into cells. Negative control shRNA Plasmid-A (cat. #sc-108,060) encodes a scrambled shRNA sequence that will not lead to degradation of any known cellular mRNA. All plasmid constructs (experimental and controls) and the transfection reagent Polybrene (Cat. # sc-134,220) were purchased from Santa Cruz Biotechnology, Inc. (Santa Cruz Biotechnology, CA, USA). Data sheet of the sequences of respective shRNA vector plasmid are available at Santa Cruz website.

### MMP20/DSPP shRNA lentiviral mediated transduction of OSC2 cells

A day prior to transfection, 5X10^5^ logarithmically growing and healthy OSC2 cells were split into six equal groups, each plated in 6-well plates in antibiotic-free DMEM/F12 media supplemented with 10% serum (Mediatech Inc. VA) to achieve a 70–80% confluence overnight. The groups were “medium only”, “Control shRNA Plasmid-A” (scrambled sequence), “copGFP Control Plasmid”, and the three experimental Plasmid groups: DSPP-shRNA, MMP20-shRNA, and combined DSPP-MMP20-shRNA. Transient transfection was carried out following the manufacturer’s protocol. Prior to transfection, cells were washed with shRNA transfection medium before adding 2 ml of medium containing 5 μg/ml Polybrene (cat. # sc-134,220) to each well. Thereafter, 30 μl (30X10^4^ particles) of lentiviral particles, equivalent to multiplicity of infection factor (MOI) 1, were added drop-wise to corresponding well and incubated overnight under normal cell culture conditions.

### Establishment of MMP20, DSPP, MMP20-DSPP stable lines

Stable lines of lentiviral-transduced shRNA cells were carried out via puromycine selection as previously described [20]. Briefly, selection of OSC2 cells stably expressing MMP20-, DSPP, MMP20DSPP-shRNA, and the Control shRNA Plasmid-A commenced 72 h post-transfection following the manufacturer’s instructions (Santa Cruz Biotechnology, Santa Cruz CA). Growth medium was aspirated from the cells and replaced with fresh selection medium containing 3 μg/mL of puromycin (cat # sc-108,071; Santa Cruz Biotecnology). Puromycin-containing medium was replaced every 2–3 days with freshly prepared selection medium, and selection of stable cells was completed in approximately 4 weeks. Stable cells were expanded, harvested, and prepared for western blot and quantitative real-time PCR (Q R-T PCR) analyses.

### Cisplatin treatment

We previously reported that treatment of OSC2 cells with various concentrations of cisplatin (5-100μΜ) resulted in correspondingly varied apoptotic rates [20]. In the present study, cisplatin (Cis-diammineplatinum II dichloride, DDP; Sigma-Aldrich, St. Louis, MO) was dissolved in 0.9% sodium chloride to achieve a stock concentration of 3.33 mM. Parent OSC2, scramble, and silenced OSC2 cells were grown in 6-well plates until 70% confluence was attained. Cisplatin was then added at concentrations of 5, 10, and 50 μM for 72 h before harvesting for Western blot assay and analysis. Based on the results of preliminary titration MTT experiments with OSC2 cells treated with 5, 10, and 20 μM concentrations of cisplatin, treatment with 10 μM of cisplatin for 72 h was selected as optimal for flow cytometry experiments.

### Western blot analysis

After washing twice with ice-cold PBS, cells were lysed in radioimmunoprecipation assay buffer (50 mM Tris pH 7.4, 150 mM NaCl, 1% Triton X-100, 1% deoxycholic acid sodium salt, 0.1% sodium dodecyl sulfate, 100 mg/ml phenylmethylsulfonyl fluoride, 1 mg/ml aprotinin, 1 mM dichlorodiphenyltrichlo-roethane and 1 mM sodium orthovanadate; Abcam, MA, USA cat#156034) for 10 min at 4 °C. Recovered lysed products were centrifuged at 40,000 x g for 15 min at 4 °C, and protein concentration measured using the Bio-Rad Protein Assay (Bio-Rad Laboratories, Inc., Hercules, CA, USA), according to the manufacturer’s protocol. Thereafter, proteins in the total cell lysate were separated by SDS-PAGE (10% separation gel and 5% spacer gel) and electrotransferred to polyvinylidene difluoride films (Bio-Rad Laboratories, Inc., Hercules, CA, USA). Blots were placed in blocking solution for 1 h at room temperature before probing with primary antibodies [rabbit polyclonal ABCG2 (1:400) and BMI1 (1:400) and rabbit monoclonal ALDH1 (1:300) all from Cell Signaling (Cell Signaling, Beverly, MA, USA cat no. #4477, #2830, #12035 respectively); mouse monoclonal PDPN / gp36 (1:300), rabbit monoclonal LRG4 (1:300), rabbit polyclonal CD133 (1:300), and rabbit monoclonal CD44 (1:250), all from Abcam (Abcam, Cambridge MA cat no. #10288, #137480, #19898, #51037 respectively] overnight at 4 °C. Blots were washed, incubated with goat polyclonal anti-rabbit IgG horse radish peroxidase secondary antibody (1:3.000; Santa Cruz Biotechnology, Santa Cruz, CA, USA, # sc-2301) or anti-mouse IgG antibody (dilution, 1:3.000; Santa Cruz Biotechnology, CA, USA, # sc-2031) at room temperature for 1 h at 25 °C. β-actin was used as control (Santa Cruz Biotechnology, Santa Cruz, CA, USA, # sc-47,778). Proteins were visualized using an enhanced chemiluminescence system and band intensity was quantified using Image J software1.48 (http://rsb.info.nih.gov/ij/). After Western blot normalization and densitometric analysis, the results were presented as percentage of the levels of each marker in either parent OSC2 (unsilenced) cells or control-scramble cells (set as 100%), depending on the experiment.

### Flow cytometry analysis with ABCG2 and ALDH1 mAb staining

Freshly harvested parent OSC2, control scramble (shC), silenced DSPP (shD), silenced MMP20, (shM), or combined silenced DSPP-MMP20 (shDM) OSC2 cells were used for ABCG2 and ALDH1 staining analysis and cell sorting by flow cytometry using a functional-grade purified anti-human ABCG2 mAb (eBioscience, San Diego; clone 5D3) and a purified anti-human ALDH1 mAb (BD Transduction Laboratories, # 611194). Briefly, all categories: OSC2; OSC2 + cisplatin; shC + cisplatin; shD + cisplatin; shM + cisplatin; and shDM+cisplatin at 0.5X10^6^ cells/100 μl were incubated with ABCG2 (1:50 dilution) or ALDH1 (1:50) antibody for 30 min on ice. Samples were then washed with PBS containing 1% FBS, and incubated with Alexafluor-488 conjugate goat anti-mouse secondary antibody (1:100**;** R&D Systems, Inc. **#** IC0041G) for 30 min. Thereafter, samples were washed with PBS and analyzed using a FACS Calibur flow cytometer (BD Biosciences). Gates in the right angle scatter versus forward scatter diagrams were used to exclude debris. At least 100,000 events were collected before analysis. All flow cytometric data were analyzed with BD Cell Quest Pro software (BD Biosciences).

### Statistical analyses

Results of protein expression levels, cell viability, and cell number of treated cells were compared with the results of untreated (control) cells. Statistical analysis was performed using statistical Packages for the Social Sciences (SPSS). One way ANOVA has been applied for the comparison of multiple groups, followed by post – hoc pairwise comparisons with the application of Dunn’s test and *statistical significant level was set* at 5% (*p* < 0.05). All experiments were performed in triplicate.

## Results

### Generation of stable DSPP***-***MMP20 silenced OSC2 cell lines

**Fig. 1:**
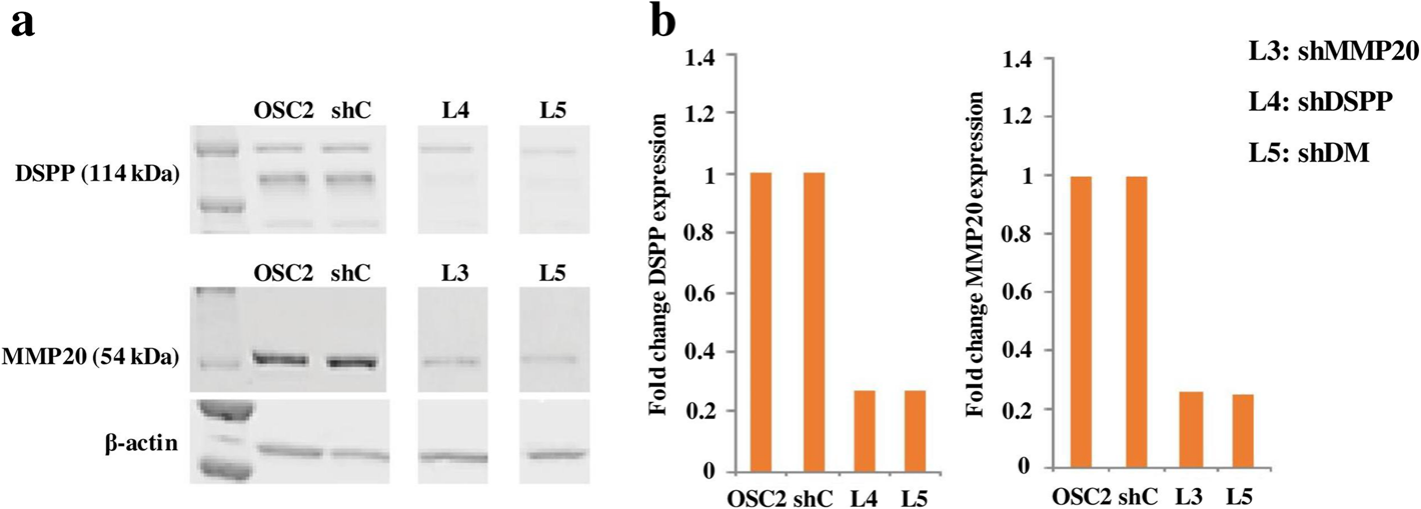
Lentiviral mediated knockdown of DSPP (shDSPP), MMP20 (shMMP20) and DSPP+MMP20 (shDM) in OSC2 cells, and generation of stable DSPP / MMP-20 / DSPP+MMP20 silenced OSC2 cell lines*.* Protein expression levels of DSPP and MMP20 (**a**) are presented as relative fold change levels in knockdowns (shDSPP, shMMP20 and shDM) compared to scramble-control (shC) (**b**). Among the different stable lines selected, L4 exhibited the maximum knockdown (73%) for DSPP-silencing, L3 for MMP20 (74%) and L5 for DSPP+MMP20 silencing (73 and 75% for DSPP and MMP20, respectively), as compared to shC at the protein level

As shown in Fig. 1, the effectiveness of lentiviral-mediated silencing of DSPP, MMP20, and combined MMP20-DSPP in OSC2 cells was verified by western blot (WB) analysis of five stable line selections (data not shown) for each of DSPP, MMP20, and MMP20/DSPP silencing. In each case, lines exhibiting the highest knockdowns: L4 (78%) for DSPP; L3 (79%) for MMP20; and L5 (73%) for combined MMP20-DSPP were selected for subsequent experiments as described above.

### CSC marker expression in parent and DSPP/MMP20 silenced cells OSC2 cells

**Fig. 2:**
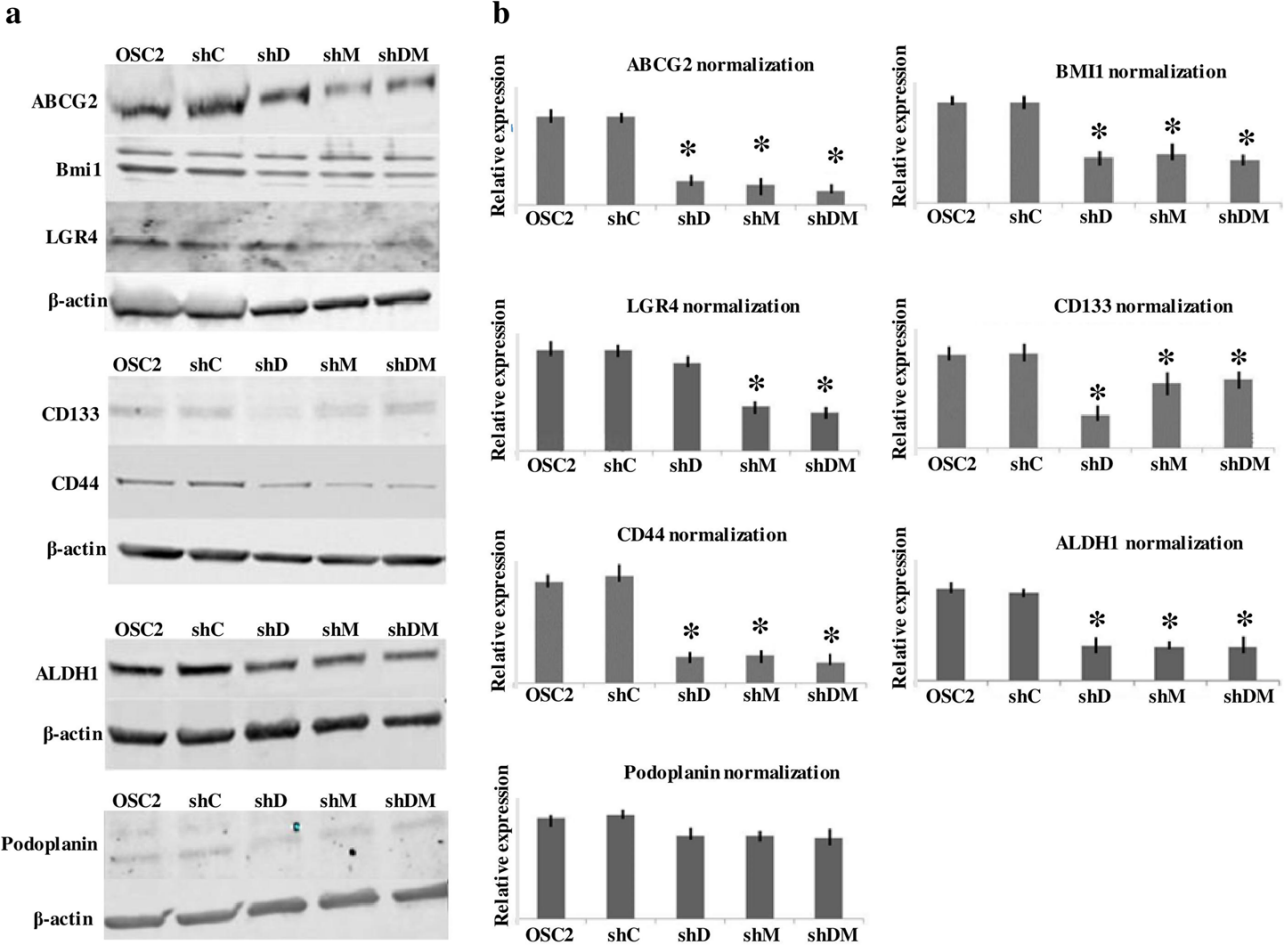
Western blot (WB) analyses of OCSC markers in DSPP-MMP20 silenced OSC2 cells (**a**). Normalization of ALDH1, ABCG2, BMI1, Podoplanin (PDPN), CD44, CD133 and LGR4 protein levels was with β-actin . Y- (vertical) axis of histograms represent expression level for each protein relative to β-actin (**b**). OSC2 = parent (untreated) cells; shC = control (scramble sequence) cells; shD = DSPP silenced cells; shM = MMP20 silenced cells; shDM = combined DSPP-MMP20 silenced cells. *p < 0.05 compared with ShC. Experiments were performed in triplicate

We first evaluated the protein expression of seven previously characterized CSC markers (ALDH1, ABCG2, BMI1, PDPN, CD44, CD133, LGR4) in OSC2 cells. Western blot revealed that all seven markers were expressed in OSC2 cells. Densitometric analysis showed higher protein levels for ALDH1, LGR4, BMI1 and CD44 (Fig. 2; see also Additional file 1: Table S1). We then assessed the effects of silencing DSPP (shD), MMP20 (shM), and combined DSPP-MMP20 (shDM) on the expression of CSC markers. As shown in Fig. 2 (WB), protein expression levels of all studied markers were significantly reduced in shD, shM, and shDM cells, compared to scramble (shC) controls (Fig. 2; see also Additional file 1: Table S1; *p < 0.05). ABCG2 and CD44 showed the most percentage downregulation in all silenced cells categories (84 and 81%, respectively, in shDM, compared with shC). On the other hand, PDPN and CD133 demonstrated less pronounced downregulations (24 and 27%, respectively, of the corresponding levels in shC cells) following combined shDM silencing (Fig. 2; see also Additional file 1: Table S1).

When the effects of DSPP versus MMP20 silencing were compared, ALDH1, ABCG2, BMI1, PDPN and CD44 demonstrated comparable percentage decreases in DSPP and MMP20 silencing. Double silencing (DSPP-MMP20) resulted in further downregulation of ABCG2 and CD44 (84 and 81%, respectively), and, to a lesser extent, BMI1 and PDPN (58 and 24%), respectively, without inducing any demonstrable additional changes in ALDH1 (62%) (Fig. 2; see also Additional file 1: Table S1). In contrast, CD133 showed higher percentage downregulation (65%) in shD compared to both shM (31%) and shDM silenced cells (27%; See also Additional file 1: Table S1). LGR4 demonstrated the highest reduction in shM (56%) and shDM (62%) silenced cells, compared with minimal downregulation (13%) in shD silenced cells (Fig. 2; see also Additional file 1: Table S1). Collectively, these results validate the presence of CSCs in OSC2 cells, and suggest that DSPP and MMP20 may regulate CSC levels.

### Cisplatin effects on CSC markers expression in DSPP/MMP20 silenced OSC2 cells

**Fig. 3:**
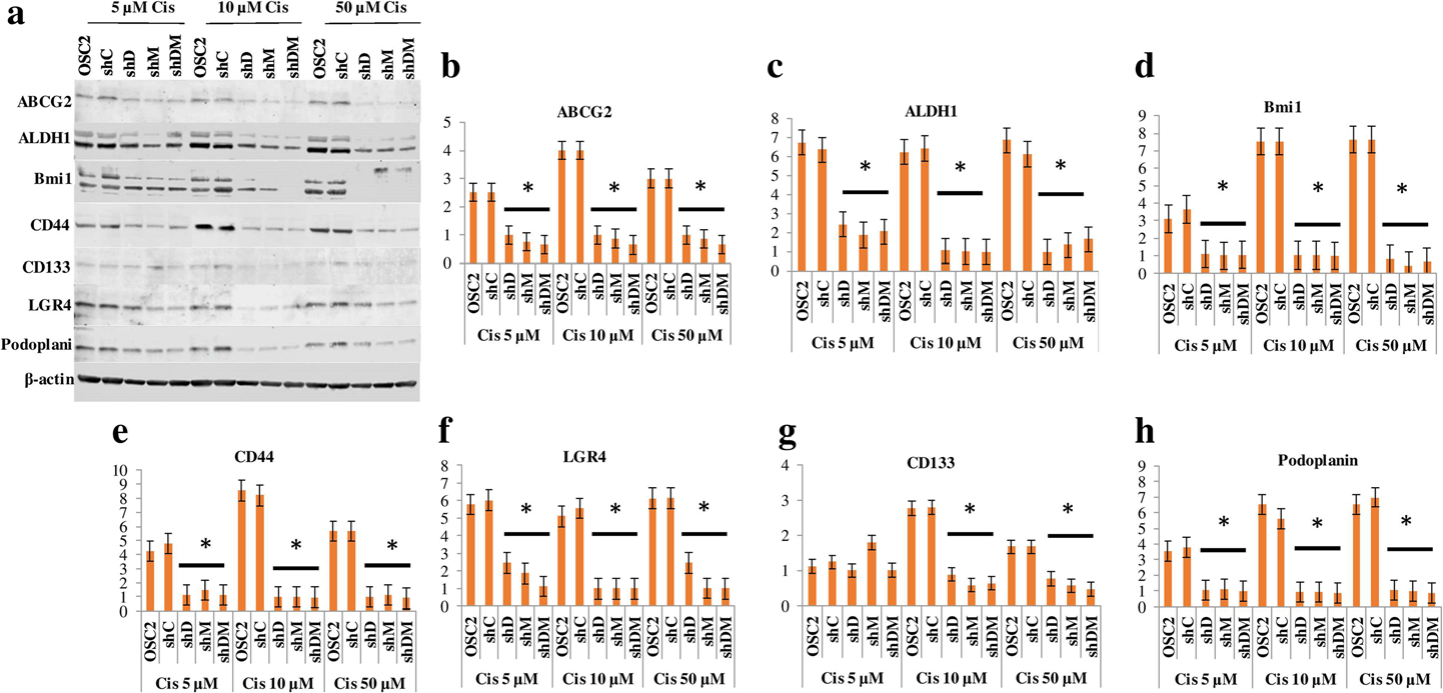
WB analysis of OCSC makers following DSPP-MMP20 silencing and treatment with Cisplatin. **a** Total protein levels; **b** ABCG2; **c** ALDH1; **d** BMI1; **e** CD44; **f** LGR4; **g** CD133; **h** Podoplanin. Treatment with cisplatin was for 72 h at concentration of 5, 10, or 50 μΜ. Normalization of protein level was with β-actin. Y-(vertical) axis of histograms represent expression level for each protein relative to β-actin. OSC2 = parent (untreated) cells; shC = control (scramble sequence) cells; shD = DSPP silenced cells; shM = MMP20 silenced cells; shDM = combined DSPP-MMP20 silenced cells. *p < 0.05 compared with ShC. Experiments were performed in triplicate

Next, we investigated the effects of DSPP/MMP20 silencing on CSC markers sensitivity to cisplatin treatment. Parent and silenced OSC2 cells (shD, shM, shDM) were treated with different dosages (5μΜ, 10μΜ, 50μΜ) of cisplatin for 72 h before assessing the effects of treatment on CSC markers. Following Western blot analysis, cisplatin treatment resulted in upregulation of most of the markers in parent OSC2 cells with the highest noted with BMI1, ALDH1, and ABCG2, at high cisplatin concentrations. PDPN, CD133, and LGR4 showed modest upregulations after cisplatin treatment of parent OSC2 cells. In contrast, CD44 was decreased in parent OSC2 cells (see Additional file 2: Table S2). On the other hand, CSC markers were significantly downregulated in ShD, ShM, and ShDM silenced OSC2 cells compared with levels in ShC (scrambled) OSC2 cells following treatment with all three concentrations of cisplatin (Fig. 3; *p < 0.05). For example, ShDM silencing resulted in downregulation of all CSC markers ranging from 19 to 82% following treatment with cisplatin at 5 μM, 77 to 88% at 10 μM, and 72 to 94% at 50 μM (Fig. 3; see also Additional file 3: Table S3).

Overall, there was no marked differences in percentage changes of CSC makers amongst the various silencing categories (shD, shM, shDM) for each cisplatin concentration used. As noted however shDM cells treated with cisplatin showed enhanced downregulation of LGR4 (82%) and PDPN (94%) for cells treated with 5 μM and 50 μM of cisplatin, respectively (Fig. 3; see also Additional file 3: Table S3).

### ABCG2 and ALDH1 positive cell population in DSPP/MMPS20 silenced cells following cisplatin treatment

**Fig. 4:**
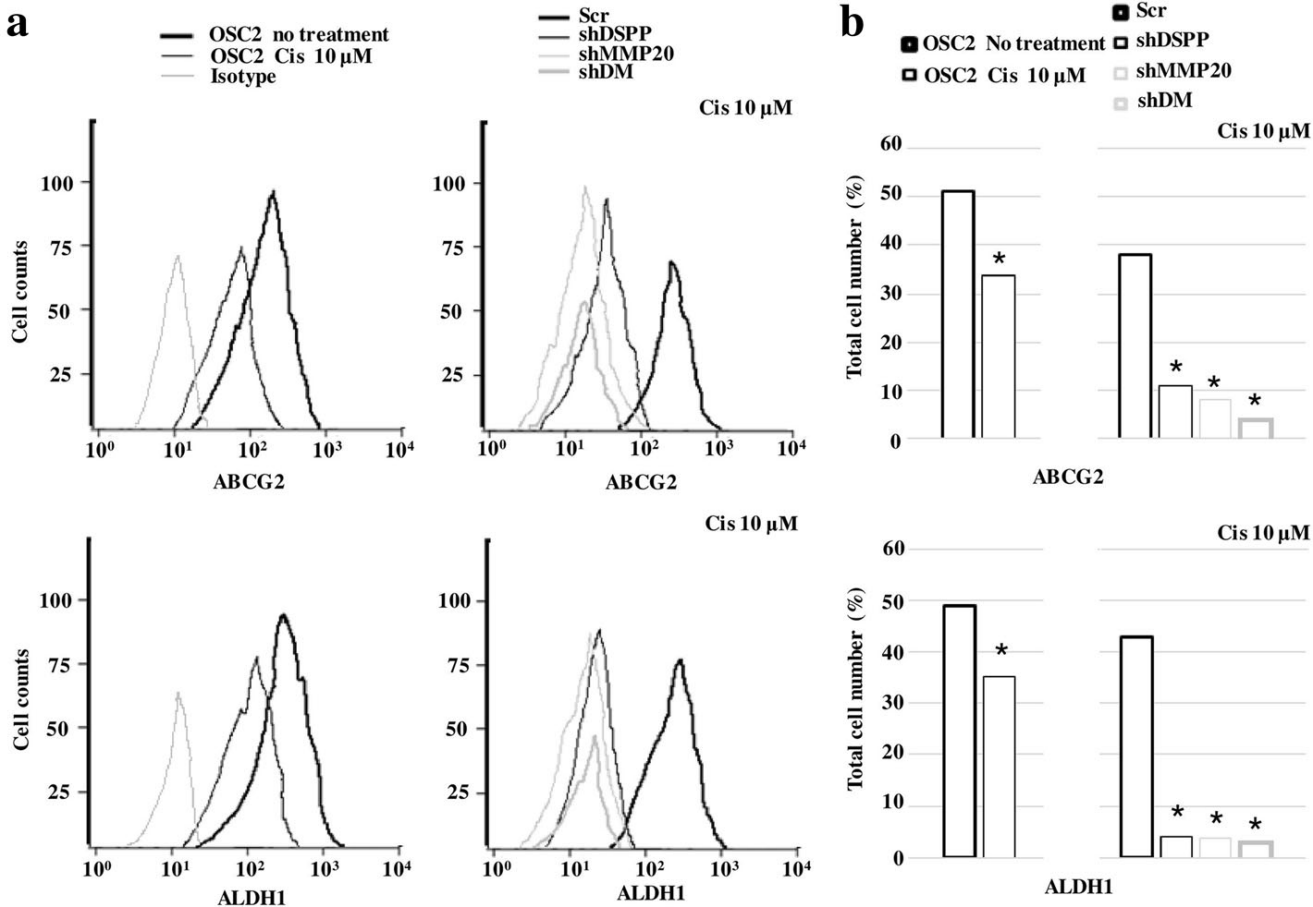
Flow cytometry analysis of ABCG2 and ALDH1 positive cell population following cisplatin treatment. Treatment with cisplatin was for 72 h at a concentration of 10 μM. **a** Representative flow cytometric histograms of ABCG2 and ALDH1 expression. Samples were analyzed after exposure to primary antibodies, and an Alexafluor-488 conjugated secondary antibody. Total cell count is depicted on the y-axis. *Left*: Isotype control antibody alone (light grey), OSC2 (untreated) cells (heavy black), and OSC2 treated (light black). *Right*: Scr = control (heavy black), shDSPP silenced cells (light black), shMMP20 silenced cells (light grey) and shDM combined DSPP-MMP20 silenced cells (heavy grey). All histograms show the distribution of fluorescence intensity (x-axis), reported as arithmetic means. **b** Quantification of ABCG2 and ALDH1 positive OSC2 cell population, presented as percentage (%) of total cell number. Statistically significant lower percentages (*p < 0.05) of ABCG2 and ALDH1 positive cell were noted in cisplatin-treated compared with untreated OSC2 cells, as well as in shDSPP, shMMP20 and shDM cells compared to scramble control (Scr) cells, respectively. Experiments were performed in triplicate

In order to corroborate our data on the effects of DSPP/MMP20 silencing on CSC markers in cisplatin-treated OSC2 cells, flow cytometric analysis was carried out to determine the percentage of ABCG2 and ALDH1 positive cells. ABCG2 and ALDH1 were chosen for flow cytometric analysis because they exhibited the highest and lowest levels, respectively, amongst the CSC markers investigated in parent OSC2 cells (Additional file 1: Table S1). As shown in Fig. 4, parent OSC2 cells showed only a modest decrease in ABCG2 (34%) and ALDH1 (35%) following cisplatin treatment, whereas ShD, ShM, and ShDM showed marked decrease in ABCG2 (6, 1, and 1%, respectively) and ALDH1 (< 5) positive cell population, compared with shC controls, following cisplatin treatment. These data further suggest that DSPP/MMP20 silencing sensitizes OSC2 cells to cisplatin treatment by decreasing CSC population in OSCC cells.

## Discussion

The present study was prompted by increasing recognition of the importance of CSCs in the development and progression of OSCCs, which constitutes an overwhelming majority of head and neck squamous cell carcinomas. Particularly, deciphering their role in chemotherapeutic resistance commonly seen in OSCC patients remains an area of intense interest [5, 6]. Our current study showed that OCSC marker CD44, CD133, ABCG2, ALDH1, PDPN, BMI1 and LRG4 are expressed in OSCC cell line. Significantly, not only did MMP20/DSPP silencing in OSCC cell line, OSC2, result in decreased OCSC markers, silencing also increased overall sensitivity of OSC2 cells (presumably its OCSCs population), to cisplatin. This provides evidence for a potential role for DSPP and MMP20 in OCSC sustainability in, and treatment resistance of, OSCCs. Following DSPP/MMP20 downregulation, ALDH1, ABCG2, BMI1, CD133, and CD44 showed similar levels of downregulation, whereas CD133 and LGR4 showed a more selective responsiveness to DSPP or MMP20 silencing, respectively. Furthermore, the levels of DSPP and MMP20 silencing-induced downregulation differed amongst CSC markers, with ABCG2 and CD44 showing more pronounced downregulations. Combined MMP20-DSPP silencing induced additional downregulation only in selected CSC markers.

Several previous studies have shed light on the expression and biologic role of CSC markers in HNSCC. ABCG2 is frequently expressed in HNSCC and, as has been determined in other cell types, its activity may be associated with stem cell proliferation and maintenance [7, 24]. In OSCC, oral precancerous lesions (OPL), and their corresponding cell lines, increased density of an ABCG2 (and BMI1] positive population has been reported [25]. In addition to its usefulness as a CSC marker, ABCG2 may be directly involved in cancer-related pathways and functions. For example, Huang et al. [26] reported that ABCG2 coexpression with V-ATPase, a cell membrane ATPase produced in microsomes and contributing to drug tolerance, positively correlated with pathologic grade and enhanced metastasis, invasion, and drug resistance in esophageal SCC. *c-Met* or hepatocyte growth factor receptor (HGFR) has also been correlated with ABCG2 expression. Indeed, c-Met knockdown decreased the expression of ABCG2 in HNSCC [27].

ALDH1 and CD44 also have been cited as reliable CSC markers in HNSCC. Chen et al. [28] reported that an enriched population of ALDH1-positive CSCs isolated from HNSCC cell lines formed three-dimensional spheroids in culture displaying epithelial mesenchymal transition characteristics, accompanied by increased ability of colony formation and invasiveness. Prince et al. [13] also showed that a small population of CD44-positive cancer cells, representing less than 10% of primary HNSCC cells, demonstrated high self-renewal and differentiation ability in an immunodeficient mouse HNSCC study model. Furthermore, Davis et al. [29] reported that HNSCC cells with high CD44 and ALDH1 positive cells displayed enhanced capacity to colonize the lungs after tail-vein injections in a HNSCC (tongue) orthotopic mouse model. Chinn et al. [12] study extended and corroborated the results of these studies by demonstrating that overexpression of CD44 alone or in combination with high ALDH1 activity in HNCSCs increased the potential for regional metastasis and propensity for primary tumor occurrence. The authors therefore concluded that the activities of CSCs are essential for invasion and regional metastasis in HNSCC tumors [12].

Other previously identified CSC markers, such as BMI1, PDPN, and CD133, were also detected in the OSC2 cell line population we investigated. In previous studies, BMI and PDPN were found to be expressed in the majority of HNSCC tumor samples, especially at the invasive front [30]. Furthermore, co-expression of the two molecules correlated with decreased overall survival associated with a decreased chemo/radio therapeutic treatment response [30]. Chen et al. [28] suggested that BMI1 contributed to the enhanced proliferative potential of laryngeal CSCs, while suppressing CSC sensitization to chemoradiotherapy. PDPN expression in circulating tumor cells was also considered as a poor prognostic factor in patients with locally advanced or metastatic HNSCC [31].

With respect to CD44 and CD133, the study by Mannelli et al. [32] showed that 93.1% of primary and lymph node metastasis of HNSCCs expressed CD44 whereas only 10.34% expressed CD133, suggesting a role for CD44 in regional lymph node spread. It is noteworthy that CD133 has long been characterized as a CSC marker in laryngeal SCC [33], while a subpopulation of CD133 positive cells has been implicated in the chemoresistance to placlitaxel in the treatment of OSCC [34]. Our current study showed a high level of LGR4 in OSC2 cells. LGR4 has been suggested as a potential global marker of adult stem cells [8], and recently was shown to serve as a critical factor for skin carcinogenesis by mediating MEK/ERK and Wnt/β-catenin pathways [35]. However, its possible role and significance in HNSCC is yet to be deciphered.

Beyond identifying CSCs in tumor samples and correlating with several diagnostic and prognostic parameters, enhanced understanding of the molecular function-mechanisms governing their sustainability is necessary to fashioning effective strategies for overcoming CSC-mediated HNSCC cell resistance to chemotherapy. Our current data reflecting an overall reduction in the population of CSC-marker positive OSC2 cells following MMP20/DSPP silencing may suggest reduced tumorigenic profile in DSPP/MMP20 silenced in OSCCs. While details of the function-mechanisms of DSPP/MMP20 silencing-induced CSC downregulation in OSCC must await the results of on-going studies, it is reasonable to speculate that potential mechanisms may implicate several oncogenic pathways, including common signaling pathways and molecules already implicated in CSC biology. For example, the antioxidant enzyme Peroxiredoxin II regulates VEGF signaling in liver CSCs by upregulating BMI1 and Sox2 through a VEGFR-2/STAT3 pathway [36], while VEGF and VEGF receptor neuropilin-2 signaling involved a BMI1-mediated mechanism in aggressive prostate cancer [37].

It also has been proposed that CD133 directly interacts with VEGF to promote cell growth, survival, and angiogenesis in both primary endothelial and melanoma cells [38]. With respect to ALDH1, a possible association between ALDH1 expression and EGFR upregulation has been suggested. Interaction between ALDH1 and EGFR in high-grade serous ovarian cancer positive for both molecules correlated with poor survival [39]. Similarly, Nalwoga et al. [40] reported an association between ALDH1 p53, and EGFR expression in aggressive breast cancer. In HNSCC, CSC marker expressions have been associated with tumor behavior. For example, PDPN was shown to induce extracellular matrix degradation and tumor invasion in association with MT1-MMP and Rho GTPases in OSCC [41]. In another study, an aggressive basal-cell-like cellular compartment of HNSCC, expressing the CSC markers CD44, ALDH1 and CK14, was correlated with MMP9 expression and invasiveness [42].

On the bases of these numerous reports linking CSC marker expression with various aspects of cancer biology, it is therefore reasonable to speculate that the changes observed with OCSC following DSPP/MMP20 silencing may be translatable into several anticancer effects. Our previously published reports show that DSPP is significantly upregulated in poorly differentiated OSCC [17]. Significantly, DSPP silencing was shown to reduce MMP2, MMP3, MMP9, p53, Ki-67, EGFR, and VEGF expression in DSPP-silenced OSC2 cells [20]. Furthermore, there was significant alterations in notable hallmarks of oral carcinogenesis, including cell cycle arrest, reduced cell viability, and reduced colony-formation ability, reduced migration and invasion [20]. More recently, we also demonstrated that DSPP interacts with MMP20 in OSCC cells [23].

The significant downregulation of CD44 expression in MMP20-silenced cells suggests a closer association, and possibly potential interaction, between the two molecules, similar to that previously described between CD44 and other MMP members. For example, MMP9 enhances CD44 cleavage and shedding, and CD44-mediated cell migration in glioblastoma xenograft cells [43], while CD44 regulates hyaluronan-dependent MMP2 secretion and tumor invasiveness in human lung carcinoma cells [44]. Zarrabi et al. [45] also suggested a crosstalk signaling between MMP14 and CD44, leading to EGFR phosphorylation, and activation of downstream MAPK and PI3K signaling pathways regulating cell migration.

Cisplatin, a first-generation anticancer drug is widely used to treat OSCC and other head and neck cancers [46]. However, treatment failures with cisplatin is very common due to rapid development of chemoresistance [47]. Mechanisms of cisplatin resistance in OSCC are yet to be fully understood. In the present study, we also investigated the effect of DSPP/MMP20 silencing on OCSC markers when treated with cisplatin. While treatment of parent OSC2 cells with cisplatin resulted in further upregulation of CSC markers, notably, BMI1, ALDH1, and ABCG2, cisplatin treatment of DSPP/MMP20 silenced cells resulted in downregulation of all CSC markers investigated. Furthermore, flow cytometry results showed a significant decrease in the population of ABCG2 and ALDH1 positive cells of cisplatin-treated DSPP/MMP20 silenced cells. These results suggest a cisplatin-induced resistant mechanism via the upregulation of CSC markers in OSCC cells. From a chemotherapeutic standpoint, these data suggest that DSPP and MMP20 silencing (alone or in combination) sensitizes OSCC cells to cisplatin treatment, possibly, by targeting CSC fractions. Indeed, our published studies show that DSPP-silenced OSC2 cells exhibited increased sensitivity to cisplatin-induced apoptosis in tumor cells [20].

Other studies have shown that cisplatin enhances BMI1 expression and increase in the stem cell fraction in a xenograft HNSCC model [48]. In HNSCC xenograft studies, the authors also demonstrated a cisplatin-induced increase in the fraction of ALDH/CD44 positive cells. [48]. The authors further showed that cisplatin treatment promoted CSCs self-renewal and survival in vitro, along with upregulation of BMI1 and Oct-4 expression in an IL-6-dependent manner [48]. Subsequent studies by Zhou et al. [49] provided evidence that podocalyxin-enhanced cisplatin chemoresistance in OSCC is mediated through the upregulation of BMI1 by a FAK-dependent promotion of BMI1 mRNA stability.

The development of chemotherapeutic strategies for overcoming cisplatin resistance in HNSCC continues to receive the attention of several investigators in the field. For example, YM155, a survivin inhibitor, has been shown to reverse cisplatin resistance in cell lines and in a xenograft model of HNSCC [50]. More recently, YMGKI-2, an active component of Antrodia cinnamomea Mycelia, was shown to abrogate ALDH activity, and to abolish cancer-initiating cell properties in HNSCC, through dual STAT3 and Src inactivation [51]. The authors also showed that YMGKI-2 treatment of cancer cells promoted cell differentiation, attenuated in vivo tumorigenicity and, notably, restored chemosensitivity in HNSCC cells [51]. Interestingly, Zhao et al. [52] showed that siRNA-mediated ABCG2 knockdown in tongue OSCC cells increased responsiveness to cisplatin, and reduced the migratory/invasive potential of the cancer cells. The authors also showed that ABCG2 is a direct target of miR-222, allowing for the possibility that miR-222 mimics will downregulate ABCG2 expression and enhance sensitivity to cisplatin.

In summary, our present report provides evidence that several CSC markers are highly upregulated in OSCC cell line, OSC2, and that MMP20/DSPP silencing resulted in their downregulations. The presence of these markers also validates the presence of CSCs in the OSCC cell line, OSC2. The increased sensitivity to cisplatin treatment shown by DSPP/MMP20-silenced OSC2 cells as well as the decreased population of OCSCs following cisplatin treatment suggest that MMP20 and DSPP contributes to the development, progression, and the response of OSCC to chemotherapy. Although our current study was limited to the use of a single oral cancer cell line, OSC2, our results provide the foundation for on-going and future function studies utilizing multiple cell lines to investigate OCSC fractions, density, and other properties in OSCCs. Future studies would be necessary for a complete understanding of the precise role and mechanisms of DSPP/MMP20 in oral carcinogenesis. For instance, rescue experiments could assess whether transfection of MMP20 and/or DSPP expression vectors to corresponding shRNA stable clones would also result in reconstitution of CSC marker expression. This is with a view to deciphering therapeutic strategies necessary to overcoming chemoresistance in OSCC and other head and neck cancers.

## Additional files

Additional file 1: **Table S1.** Protein expression levels (densitometric data after Westen blot normalization) for each studied cancer stem cell marker in parent and silenced (for DSPP, MMP20 or both) OSC2 cells. (DOC 49 kb)

Additional file 2: **Table S2.** Changes in protein expression levels for each studied cancer stem cell marker following treatment of OSC2 cells with various cisplatin concentrations (5, 10, 50 μM) for 72 h. Data are presented as percentage of the levels of each marker in control untreated cells (set as 100%) after Western blot normalization. (DOCX 16 kb)

Additional file 3: **Table S3.** Changes in protein expression levels for each studied cancer stem cell marker after 5, 10 and 50 μM of cisplatin treatment following silencing of DSPP, MMP20 or both. Data are presented as percentage of the levels of each marker in control-scramble (ShC) cells (set as 100%) after Western blot normalization. (DOC 41 kb)

## Abbreviations

ABCG2: ATP b inding cassette transporter 2
ALDH: Aldehyde dehydrogenase
BMI1: B cell-specific Moloney murine leukemia virus integration site 1
CSCs: Cancer stem cells
DSPP: Dentin sialophosphoprotein
HNSCC: Head and neck squamous cell carcinoma
LGR: Leucine-rich repeat-containing G-protein-coupled receptors
MMP: Matrix metalloproteinase
OSCC: Oral squamous cell carcinoma
PDPN: Podoplanin
SIBLING: Small integrin binding ligand n-linked glycoproteins
