# Supplementary material for: DSPP-MMP20 gene silencing downregulates cancer stem cell markers in human oral cancer cells
Source: Cell Mol Biol Lett. 2018 Jul 11;23:30. doi: 10.1186/s11658-018-0096-y (PMC6040065; doi:10.1186/s11658-018-0096-y)
Supplement: Supplementary file 1 — Table S1. Protein expression levels (densitometric data after Westen blot normalization) for each studied cancer stem cell marker in parent and silenced (for DSPP, MMP20 or both) OSC2 cells. (DOC 49 kb) [file 11658_2018_96_MOESM1_ESM.doc]

**Table S1. Protein expression levels (densitometric data after Westen blot normalization) for each studied cancer stem cell marker in parent and silenced (for DSPP, MMP20 or both) OSC2 cells.**

| | ALDH1 | ABCG2 | BMI1 | PDPN | CD44 | CD133 | LGR4 |  | | --- | --- | --- | --- | --- | --- | --- | --- | | OSC2 | 5.64 | 1.50 | 3.50 | 1.24 | 5.32 | 3.50 | 5.34 | | shC | 5.24 (100%) | 1.50 (100%) | 3.50 (100%) | 1.32 (100%) | 5.64 (100%) | 3.54 (100%) | 5.34 (100%) | | shD | 2.02 (38.5%) | 0.40 (26.7%) | 1.54 (44.0%) | 1.02 (77.3%) | 1.34 (23.8%) | 1.24 (35.0%) | 4.65 (87.1%) | | shM | 1.96 (37.4%) | 0.34 (22.7%) | 1.65 (47.1%) | 1.02 (77.3%) | 1.43 (25.4%) | 2.45 (69.2%) | 2.35 (44.0%) | | shDM | 1.98 (37.8%) | 0.24 (16.0%) | 1.46 (41.7%) | 1.00 (75.8%) | 1.07 (19.0%) | 2.57 (72.6%) | 2.00 (37.5%) | |
| --- | --- | --- | --- | --- | --- | --- | --- | --- | --- | --- | --- | --- | --- | --- | --- | --- | --- | --- | --- | --- | --- | --- | --- | --- | --- | --- | --- | --- | --- | --- | --- | --- | --- | --- | --- | --- | --- | --- | --- | --- | --- | --- | --- | --- | --- | --- | --- | --- |
|  |
|  |
|  |

OSC2: parent (untreated) cells; shC: control (scramble) cells; shD: DSPP silenced cells; shM: MMP20 silenced cells; shDM: combined DSPP and MMP20 silenced cells. In parentheses, data are presented as percentage of the levels of each marker in shC cells (set as 100%).
