# Supplementary material for: DSPP-MMP20 gene silencing downregulates cancer stem cell markers in human oral cancer cells
Source: Cell Mol Biol Lett. 2018 Jul 11;23:30. doi: 10.1186/s11658-018-0096-y (PMC6040065; doi:10.1186/s11658-018-0096-y)
Supplement: Supplementary file 2 — Table S2. Changes in protein expression levels for each studied cancer stem cell marker following treatment of OSC2 cells with various cisplatin concentrations (5, 10, 50 μM) for 72 h. Data are presented as percentage of the levels of each marker in control untreated cells (set as 100%) after Western blot normalization. (DOCX 16 kb) [file 11658_2018_96_MOESM2_ESM.docx]

**Table S2. Changes in protein expression levels for each studied cancer stem cell marker following treatment of OSC2 cells with various cisplatin concentrations (5, 10, 50 μM) for 72 hours. Data are presented as percentage of the levels of each marker in control untreated cells (set as 100%) after Western blot normalization.**

|  | ALDH1 | ABCG2 | BMI1 | PDPN | CD44 | CD133 | LGR4 |
| --- | --- | --- | --- | --- | --- | --- | --- |
| Cis 5.0μM | 166% | 89% | 287% | 108% | 32% | 80% | 120% |
| Cis 10.0μM | 266% | 215% | 524% | 96% | 79% | 160% | 110% |
| Cis 50.0μM | 200% | 218% | 526% | 114% | 48% | 106% | 121% |
