# Supplementary material for: DSPP-MMP20 gene silencing downregulates cancer stem cell markers in human oral cancer cells
Source: Cell Mol Biol Lett. 2018 Jul 11;23:30. doi: 10.1186/s11658-018-0096-y (PMC6040065; doi:10.1186/s11658-018-0096-y)
Supplement: Supplementary file 3 — Table S3. Changes in protein expression levels for each studied cancer stem cell marker after 5, 10 and 50 μM of cisplatin treatment following silencing of DSPP, MMP20 or both. Data are presented as percentage of the levels of each marker in control-scramble (ShC) cells (set as 100%) after Western blot normalization. (DOC 41 kb) [file 11658_2018_96_MOESM3_ESM.doc]

**Table S3. Changes in protein expression levels for each studied cancer stem cell marker after 5, 10 and 50μM of cisplatin treatment following silencing of DSPP, MMP20 or both. Data are presented as percentage of the levels of each marker in control-scramble (ShC) cells (set as 100%) after Western blot normalization.**

| Cis5.0μM |  | ALDH1 | ABCG2 | BMI1 | PDPN | CD44 | CD133 | LGR4 |
| --- | --- | --- | --- | --- | --- | --- | --- | --- |
|  | shD | 38.1% | 39.2% | 30.4% | 26.5% | 22.9% | 80.6% | 40.7% |
|  | shM | 29.1% | 29.6% | 27.9% | 29.7% | 31.2% | 143.0% | 30.9% |
|  | shDM | 32.1% | 25.6% | 28.7% | 26.0% | 22.9% | 80.6% | 18.4% |
|  |  |  |  |  |  |  |  |  |
| Cis 10.0μM | | ALDH1 | ABCG2 | BMI1 | PDPN | CD44 | CD133 | LGR4 |
|  | shD | 16.2% | 24.2% | 13.7% | 16.6% | 12.1% | 31.2% | 18.1% |
|  | shM | 15.7% | 21.5% | 13.7% | 16.1% | 12.1% | 20.5% | 17.6% |
|  | shDM | 15.0% | 15.7% | 13.3% | 15.4% | 11.5% | 22.6% | 17.8% |
|  |  |  |  |  |  |  |  |  |
| Cis 50.0μM | | ALDH1 | ABCG2 | BMI1 | PDPN | CD44 | CD133 | LGR4 |
|  | shD | 15.8% | 32.6% | 10.7% | 14.6% | 17.6% | 44.9% | 39.9% |
|  | shM | 21.8% | 28.3% | 5.8% | 14.0% | 19.5% | 33.5% | 16.2% |
|  | shDM | 26.7% | 21.3% | 8.5% | 6.0% | 15.6% | 27.5% | 16.1% |

shD: DSPP silenced cells; shM: MMP20 silenced cells; shDM: combined DSPP and MMP20 silenced cells
